# GaborNet Visual Encoding: A Lightweight Region-Based Visual Encoding Model With Good Expressiveness and Biological Interpretability

**DOI:** 10.3389/fnins.2021.614182

**Published:** 2021-02-04

**Authors:** Yibo Cui, Kai Qiao, Chi Zhang, Linyuan Wang, Bin Yan, Li Tong

**Affiliations:** Henan Key Laboratory of Imaging and Intelligent Processing, PLA Strategic Support Force Information Engineering University, Zhengzhou, China

**Keywords:** GaborNet-VE, visual encoding model, fMRI, expressiveness, biological interpretability

## Abstract

Computational visual encoding models play a key role in understanding the stimulus–response characteristics of neuronal populations in the brain visual cortex. However, building such models typically faces challenges in the effective construction of non-linear feature spaces to fit the neuronal responses. In this work, we propose the GaborNet visual encoding (GaborNet-VE) model, a novel end-to-end encoding model for the visual ventral stream. This model comprises a Gabor convolutional layer, two regular convolutional layers, and a fully connected layer. The key design principle for the GaborNet-VE model is to replace regular convolutional kernels in the first convolutional layer with Gabor kernels with learnable parameters. One GaborNet-VE model efficiently and simultaneously encodes all voxels in one region of interest of functional magnetic resonance imaging data. The experimental results show that the proposed model achieves state-of-the-art prediction performance for the primary visual cortex. Moreover, the visualizations demonstrate the regularity of the region of interest fitting to the visual features and the estimated receptive fields. These results suggest that the lightweight region-based GaborNet-VE model based on combining handcrafted and deep learning features exhibits good expressiveness and biological interpretability.

## Introduction

Human and primate visual systems are exceedingly adept at achieving complicated vision tasks based on rudimentary visual perception. A key research goal in the study of such visual systems is to comprehensively understand the neuronal basis of vision, especially the stimulus–response characteristics of neuronal populations (Ukita et al., 2019). This goal can be achieved through using functional magnetic resonance imaging (fMRI), which can measure brain activity from human subjects passively viewing natural images (Naselaris et al., 2011; Kay, 2018). In particular, based on the blood-oxygen-level-dependent (BOLD) signals, visual encoding models can be built to predict neural responses to arbitrary stimuli and hence develop a better understanding of visual information processing in the human brain (Cadena et al., 2019). However, a key challenge in this modeling and prediction process is the difficulty of constructing an effective non-linear feature space to analyze the non-linearity of neuronal responses.

In early visual encoding models, feature spaces were mainly constructed based on non-linear handcrafted features inspired by some visual representation mechanisms. As well, previous studies showed that neurons in the early stages of cortical visual processing have smaller receptive fields (RFs) and higher sensitivity to low-level features such as texture (Hubel and Wiesel, 1962), whereas neurons further down the ventral stream have larger RFs, higher representation invariance, and stronger response to complicated shapes (Hubel and Wiesel, 1968; Hung et al., 2005). In addition, neural mechanisms in the primary visual regions could be reliably modeled by Gabor wavelets with variations in location, orientation, and spatial frequency (Adelson and Bergen, 1985; Jones and Palmer, 1987; Carandini et al., 2005). Hence, Kay et al. (2008) proposed the Gabor wavelet pyramid (GWP) visual encoding model that consists of an over-complete basis of phase-invariant Gabor wavelets with different positions, orientations, and spatial frequencies. This model processes visual stimuli to generate a non-linear feature space with good expressiveness and interpretability. Thus, the GWP model became a classical encoding model for low-level visual cues. The key aspects and prediction outcomes of the GWP model were further improved in many subsequent studies (Naselaris et al., 2009; Nishimoto et al., 2011; Vu et al., 2011; Li et al., 2018; St-Yves and Naselaris, 2018). Nevertheless, the GWP model is not suitable for higher-level visual processing. Naselaris et al. (2009) made a voxel-to-voxel comparison of natural scene category labels between the GWP model and a semantic model. The results demonstrated that although the predictions of the two models are competitive, each model excels the other. Specifically, the GWP model predictions are more accurate for voxels in the primary visual cortex, whereas the semantic model predictions show higher accuracy for voxels in the secondary visual cortex. Because high-level features are hard to design manually, the feature space is usually composed of semantic labels obtained through manual image annotation. Consequently, the voxel activity in several secondary cortex areas could be accurately predicted by semantic features (Mitchell et al., 2008; Naselaris et al., 2009, 2012; Huth et al., 2012; Stansbury et al., 2013). However, the performance of the semantic models for visual encoding is typically degraded by the inevitable subjective selection and judgment bias in the manual semantic labeling process. In summary, encoding models based on handcrafted features have strong biological interpretability but limited prediction performance and poor universality because such models are usually designed for few certain visual regions.

Recently, deep neural networks (DNNs) have made breakthroughs in a variety of domains (such as computer vision) and hence caught the attention of researchers in computational neuroscience (Kietzmann et al., 2017; Cichy and Kaiser, 2019; Richards et al., 2019; Serre, 2019). Indeed, numerous methods in neuroscience have used convolutional neural networks (CNNs) to model the human visual system and thus achieved unprecedented improvements in creating visual encoding models. Generally, these CNN-based visual encoding modeling approaches can be categorized into either task- or data-driven approaches.

On the one hand, the task-driven approaches essentially model neural responses by generating non-linear features based on an intermediate layer of a CNN pretrained on a higher vision task (Yamins and DiCarlo, 2016). This transfer learning approach has boosted the performance in many computer vision and machine learning tasks where labeled data are limited (Oquab et al., 2014). Motivated by these performance improvements, Agrawal et al. (2014) used CNN-based features to model brain activity and simultaneously predict such activity with high accuracy in the low-level, intermediate, and high-level stages of the visual pathway. Güçlü et al. used two different CNNs for layer-wise analysis of voxel scores in the ventral stream (Güçlü and van Gerven, 2015) and dorsal stream (Güçlü and van Gerven, 2017). The experimental results of these two methods reveal a gradient in the representation complexity. More recently, feature representations have been improved through novel DNN architectures, such as ResNet (Wen et al., 2018), recurrent neural networks (Shi et al., 2018), and the variational autoencoder (Han et al., 2017). However, these feature representations were not directly learned for the visual encoding task, a shortcoming that resulted in a data–task mismatch. Hence, the associated fixed features are not suitable for encoding visual cortical responses. Moreover, from a machine learning perspective, a task-driven approach has two independent linear and non-linear mapping components, and the corresponding optimization scheme might fail to reach the global optimum and instead get stuck into a local optimum.

On the other hand, the data-driven approaches directly learn all parameters of a designed encoding model based on experimental stimuli to obtain visual cortical responses. This end-to-end scheme led to successful DNN applications in many domains and thus replaced the traditional two-step machine-learning scheme. For example, Qiao et al. (2019) designed an end-to-end CNN regression (ETECR) model for visual encoding based on fMRI data. This model combined linear and non-linear mapping components into the CNN architecture, which was trained by experimental stimuli and corresponding fMRI signals. Hence, this model could learn optimal feature representations and linear regression weights for visual cortical responses and achieve major improvements in prediction performance. However, the ETECR model has limited biological interpretability because of the black box nature of its CNN underlying architecture.

Collectively, visual encoding models based on handcrafted features have good biological interpretability, but their prediction performance is limited. Alternately, visual encoding models based on CNN features exhibit excellent expressiveness but lack sufficient biological interpretability. Therefore, hybrid visual encoding models are sought to combine the biological interpretability of handcrafted features and the expressiveness of deep features. In this paper, we introduce the end-to-end GaborNet visual encoding (GaborNet-VE) model for the brain visual ventral stream. This model is trained on non-linear deep feature representations based on Gabor features (Liu and Wechsler, 2001, 2002; Kyrki et al., 2004) (to be introduced in detail in section “Gabor Filters in the First GaborNet Visual Encoding Layer”). Firstly, we designed a lightweight GaborNet-VE regression model composed of a Gabor convolutional layer, two regular convolution layers, and a fully connected layer. The Gabor convolutional layer embeds a set of learnable parametric Gabor filters. The GaborNet-VE model has an efficient region-based encoding scheme where all voxels in one region of interest (ROI) are jointly encoded. Selective optimization of features and voxels was used in our model to weigh them more effectively. In comparison with three reference models (namely, the GWP, CNN-linear, and ETECR models), our model achieved state-of-the-art prediction performance for the primary visual cortex and comparable prediction performance for the intermediate and higher cortex areas. Hence, the GaborNet-VE model has good expressiveness. Then, the guided backpropagation (GBP) algorithm was used to visualize the effective pixels and the Gabor kernels for each voxel based on the response of the top 100 best-predicted voxels in each ROI. Our visualizations showed that the proposed visual encoding model could measure the RFs of each voxel. In fact, voxels in early visual areas had smaller RFs, whereas voxels further down the ventral pathway had larger RFs. Moreover, our model could learn the preferred Gabor kernels of each voxel. The properties of these kernels demonstrated the properties of fitting to visual features of ROI. Overall, the voxels in V1 and V2 prefer Gabor kernels with high spatial frequencies, whereas the voxels in V4 and LO prefer Gabor kernels with low spatial frequencies, although special voxels with opposite properties exist in all areas. These results suggest that the lightweight GaborNet-VE model based on combining handcrafted and deep ROI features has both good expressiveness and biological interpretability. Clearly, our work connects deep learning with neuroscience and promotes the development of artificial intelligence and the understanding of human intelligence.

## Materials and Methods

### Experimental Data

To evaluate the proposed methods, a publicly available dataset (introduced in Kay et al., 2008 and Naselaris et al., 2009) was analyzed. All experimental details were presented in the studies mentioned earlier (Kay et al., 2008; Naselaris et al., 2009). Hence, we will only briefly summarize the data collection process here.

The dataset contains training and validation data of BOLD fMRI responses preprocessed after being collected from two male subjects (S1 and S2), while they were viewing natural images. The training image library included 1,750 grayscale images, each of which was presented twice. The validation image library included 120 different grayscale images, each of which was presented 13 times. Photographs were presented in successive 4-s trials; in each trial, a photograph was presented for 1 s, and the gray background was presented for 3 s. Each 1-s presentation consisted of a photograph being flashed ON–OFF–ON–OFF–ON where ON corresponds to the presentation of the photograph for 200 ms and OFF corresponds to the presentation of the gray background for 200 ms. The data collection was performed using a 4-T Varian INOVA magnetic resonance (Varian, Inc., Palo Alto, CA, United States) scanner. Eighteen coronal slices were obtained from the occipital cortex (slice thickness = 2.25 mm, slice gap = 0.25 mm, field-of-view = 128 mm × 128 mm). The BOLD signals were collected using a T2^∗^-weighted, slice-interleaved, single-shot, gradient-echo pulse sequence of echo-planar imaging (spatial resolution = 2 mm × 2 mm × 2.5 mm, flip angle = 20°, TE = 28 ms, TR = 1 s, matrix size 64 × 64).

### Framework of the GaborNet Visual Encoding Model

**FIGURE 1:**
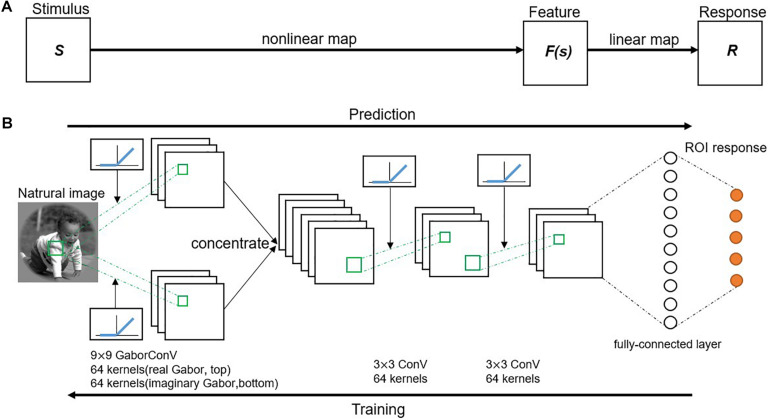
Proposed visual encoding model. **(A)** Model framework. A visual stimulus is transformed into a voxel response in two stages. First, visual stimulus (S) is transformed into a non-linear feature space [F(s)] by a non-linear mapping. Then, feature space is transformed into a voxel response (R) by a linear mapping. **(B)** A schematic diagram of the GaborNet-VE model. Response to a natural image is predicted by an end-to-end model consisting of a Gabor convolutional layer, two successive regular convolutional layers, and a fully connected layer. Gabor convolutional layer has 128 Gabor kernels with 64 kernels for each of the real and imaginary types. Size of each Gabor kernel is 9 × 9. Also, each regular convolutional layer has 64 kernels of a size of 3 × 3. Each convolutional layer is followed by a rectified linear unit (ReLU) transformation. Fully connected layer calculates weighted sum of inputs from previous layer followed by a ReLU transformation.

The GaborNet-VE model adopted a region-based encoding scheme where all voxels of one ROI in the visual cortex are jointly encoded (Zhang et al., 2019). Therefore, the GaborNet-VE model was trained by fMRI data collected from all ROIs. Model training and testing were implemented under the deep learning framework, PyTorch (0.4.0). The proposed visual encoding model consists of an input layer, a Gabor convolutional layer, several regular convolutional layers, several fully connected layers, and an output layer. The activation function of a convolutional layer is defined as the rectified linear unit (ReLU) (Nair and Hinton, 2010) transformation of a two-dimensional convolution of the activation function of the previous layer. The activation function of a fully connected layer is defined as the non-linear ReLU transformation of the weighted sum of the activation functions of the previous layer. In addition, the Gabor convolutional layer can be of a one-way real type only or a two-way combination of real and imaginary types. The number of Gabor convolutional filters in that layer is 64 or 128, and the size of each filter is (7, 7), (9, 9), (11, 11), or (13, 13). The number of convolutional filters in a regular convolutional layer is 64, and the size of each filter is (3, 3). In each convolutional layer, the stride size is (2, 2), and valid padding is used. The mini-batch size, the optimizer type [stochastic gradient descent or adaptive moment estimation (Adam) (Kingma and Ba, 2014)], the learning rate decay coefficient, and the number and types of hidden layers (Gabor convolutional, regular convolutional, or fully connected layers) were optimized with a fivefold cross-validation scheme for the fMRI data of V1 and V2. The optimized hyperparameters of GaborNet-VE are as follows: the hidden layer structure has one two-way Gabor convolutional layer and two regular convolutional layers, followed by one fully connected layer (Figure 1); the number of Gabor convolutional filters is 128 (equally divided among the real and imaginary types); the size of each Gabor filter is (9, 9); the mini-batch size is 128; the Adam optimizer is used; the learning rate decay coefficient is 0.001. All other hyperparameters were kept fixed. Moreover, GaborNet-VE used the ETECR model learning strategy with selective optimization of features and voxels. Details of this learning strategy are shown in section “Optimization Strategy.”

### Gabor Filters in the First GaborNet Visual Encoding Layer

The Gabor filters, introduced by Dennis Gabor, is a family of bandpass filters, which accept or reject inputs within a range of spatial frequencies (Gabor, 1946). These filters have been used as an efficient tool in diverse pattern analysis applications (Huang et al., 2004) for extracting different types of textures, edges, and spatially localized spectral features. Recent deep network visualization results demonstrated that Gabor-like kernels were mostly used in the first convolutional layers of CNNs trained on large-scale natural image datasets (Krizhevsky et al., 2017). The similarity between Gabor and convolutional kernels and the inherent error resiliency of deep networks represent the basis for incorporating Gabor kernels into the proposed network. Hence, a Gabor convolutional layer replaces the regular convolutional layer as the first layer in our GaborNet-VE model.

A Gabor filter is a Gaussian filter modulated by a complex sinusoidal wave (Alekseev and Bobe, 2019). This filter is monotonous and differentiable and can be defined mathematically as follows:

(1) $$g\left(x,y;\omega ,\sigma ,\varphi ,\theta \right)=\exp\left(-\frac{x^{\prime 2}+y^{\prime 2}}{2\sigma^{2}}\right)\times exp\left(j\left(\omega x^{\prime }+\varphi \right)\right),$$

(2) $$g_{rea}\left(x,y;\omega ,\sigma ,\varphi ,\theta \right)=\exp\left(-\frac{x^{\prime 2}+y^{\prime 2}}{2\sigma^{2}}\right)\times cos\left(\omega x^{\prime }+\varphi \right),$$

(3) $$g_{img}\left(x,y;\omega ,\sigma ,\varphi ,\theta \right)=\exp\left(-\frac{x^{\prime 2}+y^{\prime 2}}{2\sigma^{2}}\right)\times sin\left(\omega x^{\prime }+\varphi \right),$$

Where

(4) $$x^{\prime }=xcos\theta +ysin\theta ,$$

(5) $$y^{\prime }=-xsin\theta +ycos\theta .$$

The complex form of the Gabor filter (Eq. 1) can be decomposed into a real part (Eq. 2) and an imaginary part (Eq. 3). The center frequency of the Gabor wavelet is controlled by Eq. 4 and Eq. 5. The Gabor filter can be modified through the tuning of many parameters, which plays various roles in extracting image features. The standard deviation parameter, σ, within the Gaussian function controls the function spread. The orientation parameter within the sinusoidal wave is denoted by θ, and is used by the filter to extract features at different angles. The sinusoidal spatial frequency parameter, ω, controls the wavelength and the bar width for a Gabor wavelet. Indeed, a Gabor wavelet with a wide bar has a low ω, whereas the one with a narrow bar has a large ω. The phase shift parameter, φ, controls the sinusoidal phase offset. These parameters are initialized based on a previous study (Meshgini et al., 2012). In particular, the ω and θ parameters of the Gabor kernels are set as follows:

(6) $$\omega_{m}=\frac{\pi }{2}{\sqrt{2}}^{m},m=0,1,\ldots ,4,$$

(7) $$\theta_{n}=\frac{\pi }{8}\left(n-1\right),n=1,2,\ldots ,8.$$

The σ and φ parameters are initialized randomly from the uniform distributions U (0, 5) and U (0, π), respectively.

### Optimization Strategy

Following the ETECR model (Qiao et al., 2019), we adopt a selective optimization strategy of features and voxels in our model. To increase voxel attention to well-related features, the fully connected layer used self-adapting regression weights, a policy that is different from that of general linear regression with regularization. This dynamical learning scheme of feature weights was implemented by squaring the original weights. In Eq. 8, one stimulus image, *s*_*i*_, is transformed into the corresponding feature space (*f*_*i*_) by three convolutional layers, *Conv*().

(8) $$f_{i}=\mathrm{Conv}\left(s_{i}\right),$$

In Eq. 9, a regular weight *w*_*fc*_ is replaced by its square $w_{fc}^{2}$ in the fully connected layer, which maps the features to the predicted responses, $\hat{r_{i}}$ .

(9) $$\hat{r_{i}}=w_{fc}^{2}f_{i}+b,$$

In this way, the learning rate (μ*w*_*fc*_) of weights can be dynamically adjusted according to the current status, where μ is the original learning rate,

(10) $$\Delta w_{fc}=-\mu w_{\mathrm{fc}}\Delta \hat{r_{i}},$$

For selective voxel optimization, noise regularization, and a weighted correlation loss function were used. To reduce the influence of ineffective voxels, Gaussian noise *n*_*g*_ with zero mean and unit variance is added to each of the predicted responses $\hat{r_{i}}$ ,

(11) $$\hat{r_{i}}=\hat{r_{i}}+n_{g},$$

The Pearson correlation (ρ_*m*_ ∈ [−1,1]) of the predicted responses (*r*_*m*_) and the actual responses ($\hat{r_{m}}$) of the *m*_*th*_ voxel is calculated as

(12) $$\rho_{m}=cor\left(r_{m},\hat{r_{m}}\right)=\frac{Cov\left(r_{m},\hat{r_{m}}\right)}{\sqrt{Var\left(r_{m}\right)\cdot Var\left(\hat{r_{m}}\right)}},$$

To focus the attention of the proposed model on more effective voxels, we introduced the dynamical weights, $\eta_{m}=\rho_{m}^{2}$, in the loss function

(13) $$Loss=-\frac{\sum_{m}\eta_{m}\rho_{m}}{n}+\gamma \left|\frac{\sum_{m}\hat{r_{m}}}{n}\right|,$$

instead of computing the average ρ of all voxels in one ROI. The final form of the loss function is given by

(14) $$Loss=-\frac{\sum_{m}\rho_{m}^{3}}{n}+\gamma \left|\frac{\sum_{m}\hat{r_{m}}}{n}\right|,$$

where γ is used to control the relative contributions of the fidelity and regularization terms.

### Guided Backpropagation

With the emergence of deep network architectures, several algorithms have been proposed to visualize and interpret network outcomes. Firstly, a deconvolutional network (DeconvNet) approach was used to visualize the most discriminative image details for given neurons in network layers (Zeiler and Fergus, 2013). However, the DeconvNet visualization results are not generally clear and recognizable, especially with the large computational complexity of high-level features. An alternative visualization approach based on backpropagation (Rumelhart et al., 1986) involves computing the backward gradient of the activation function of a single unit. The main difference between the backpropagation and deconvolution approaches is the method of calculating the backward signal passing through the ReLU non-linearity. Although the backpropagation approach handles only backward values with gradients greater than 0,

(15) $$G_{i}^{l}=\left(f_{i}^{l}>0\right)\times G_{i}^{l+1},$$

the DeconvNet approach deals only with backward values greater than 0,

(16) $$G_{i}^{l}=\left(G_{i}^{l+1}>0\right)\times G_{i}^{l+1},$$

where $f_{i}^{l}$ is a bottom feature in the layer *l* from the forward direction, and $G_{i}^{l}$ is a top gradient in the layer *l* from the backward direction. The GBP visualization algorithm (Springenberg et al., 2014) combines backpropagation and deconvolution approaches and masks out both negative values corresponding to the top gradient and bottom feature,

(17) $$G_{i}^{l}=\left(f_{i}^{l}>0\right)\times \left(G_{i}^{l+1}>0\right)\times G_{i}^{l+1}.$$

The GBP algorithm enhances the performance of the ordinary backpropagation algorithm through limiting negative gradients. Such negative gradients are indeed undesirable, as they are typically associated with image regions that weaken feature visualization.

### Reference Visual Encoding Models

To further assess the performance of our proposed model, we compared it with the following three state-of-the-art visual encoding models:

#### Gabor Wavelet Pyramid Model (Kay et al., 2008)

The GWP model is a classical voxel-based visual encoding model proposed by Kay et al. (2008). This model has demonstrated superior performance in visual retrieval tasks based on fMRI. The model firstly processes stimulus images using a family of handcrafted quadrature-phase Gabor wavelets with different spatial frequencies, orientations, and locations. Then, the prediction responses are mapped linearly by the square roots of the pooled energies of the Gabor features. In our experiments, the same number of Gabor wavelets as that in Kay et al. (2008) was used on 128 × 128-pixel images, and a linear mapping was obtained by regularized orthogonal matching pursuit (Zhang et al., 2019).

#### Convolutional Neural Network-Linear Model

The CNN-linear model has several advantages, which have resulted in remarkable visual encoding performance. The model used herein follows the description in Güçlü and van Gerven (2015) and Eickenberg et al. (2016). Firstly, the feature space is composed of non-linear features obtained from each of the eight layers of the pretrained AlexNet model. Then, a linear mapping from the feature space to the brain activity space is fitted using the regularized orthogonal matching pursuit algorithm. Hence, eight voxel-based encoding models were constructed from eight feature spaces for each ROI voxel. Lastly, the model with the best prediction performance was selected as the CNN-linear encoding model.

#### End-to-End CNN Regression Model (Qiao et al., 2019)

The two encoding models mentioned earlier are linearized voxel-based models with a two-step procedure. To overcome the limitations of these models, the ETECR model was proposed. The ETECR model has four convolutional layers and one fully connected layer and is trained by an end-to-end regression scheme. The self-adapting weight learning and weighted correlation loss are used in the model construction, leading to streamlined optimization of the effective voxels. Moreover, the ETECR model encodes jointly and efficiently all voxels of one ROI. Therefore, the ETECR model simultaneously offers a lightweight architecture, effectiveness, and efficiency.

### Quantification of Model Performance

To quantify the prediction performance of each encoding model, the voxel-wise prediction accuracy is defined as the Pearson correlation (see Eq. 12) between the real and predicted responses on the validation dataset. For each model type, we carried out a replacement test to test whether the prediction accuracy of each voxel is significantly bigger than the value set by the null hypothesis. For the 120 images in the validation dataset, we randomly disturbed the correspondence between the real and predicted responses and recalculated the prediction accuracy of each voxel. After repeating this procedure 1,000 times, a null-hypothesis distribution was generated. Then, the response for one voxel can be predicted accurately when the prediction accuracy is higher than 0.27, which is significantly above the null hypothesis distribution (*p* < 0.001). Then, the replacement test was used to assess the significance of a model superiority compared with the other models. The voxels, whose responses were accurately predicted by both models, were selected for random shuffling with a 0.5 probability. According to the null-hypothesis distribution obtained by repeating the procedure mentioned earlier 1,000 times, one model significantly outperforms the other when the former can accurately predict more than 53% of the voxels (*p* < 0.05).

**FIGURE 2:**
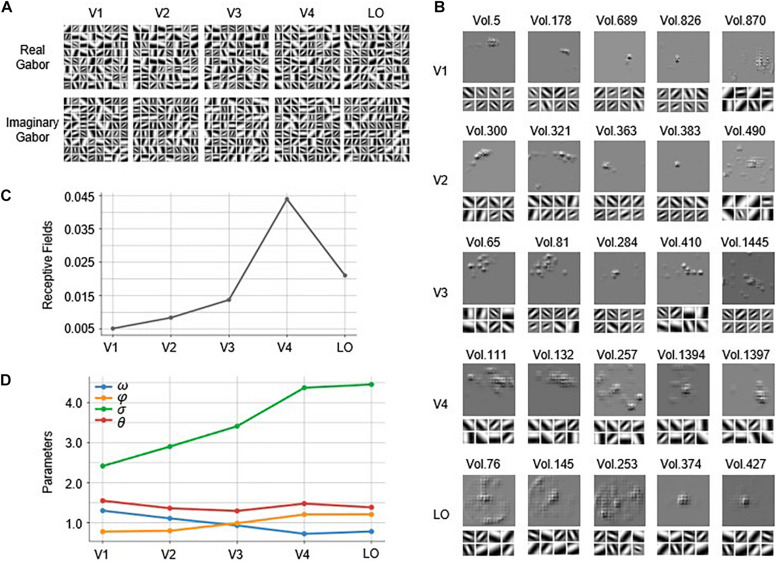
Voxel-to-voxel comparisons of GaborNet-VE, GWP, CNN-linear, and ETECR models. **(A)** Pairwise comparisons of GaborNet-VE model the GWP, CNN-linear, and ETECR models. Each of six performance plots shows a comparison of prediction accuracies for two models. Position along vertical axis indicates average prediction accuracy for models under comparison; shifts to right or left along the horizontal axis indicate a relative improvement in prediction accuracy for one of compared models. Color of each hexagonal bin indicates log-scaled number of voxels in a local region of plot. Histogram at top of each plot represents distribution of relative improvements for all voxels whose prediction accuracy is above 0.27 (*p* < 0.001, randomization test) for at least one of two models. This distribution corresponds graphically to all voxels above red dashed line. Number on each side represents fraction of voxel predictions that are improved under corresponding model. In all plots, a shift of the data toward right indicates an advantage for GaborNet-VE model, whereas a shift toward left indicates an advantage for reference models. Upper plots display data for voxels in primary visual cortex (V1, V2, and V3); lower plots display data for voxels in intermediate and higher visual areas (V4 and LO). **(B)** Joint comparisons of GaborNet-VE model and all of reference models. Each plot contains percentages of voxels in one ROI best predicted by each encoding model. Red, gray, green, and blue curves represent GaborNet-VE, GWP, CNN-linear, and ETECR models, respectively.

**FIGURE 3:**
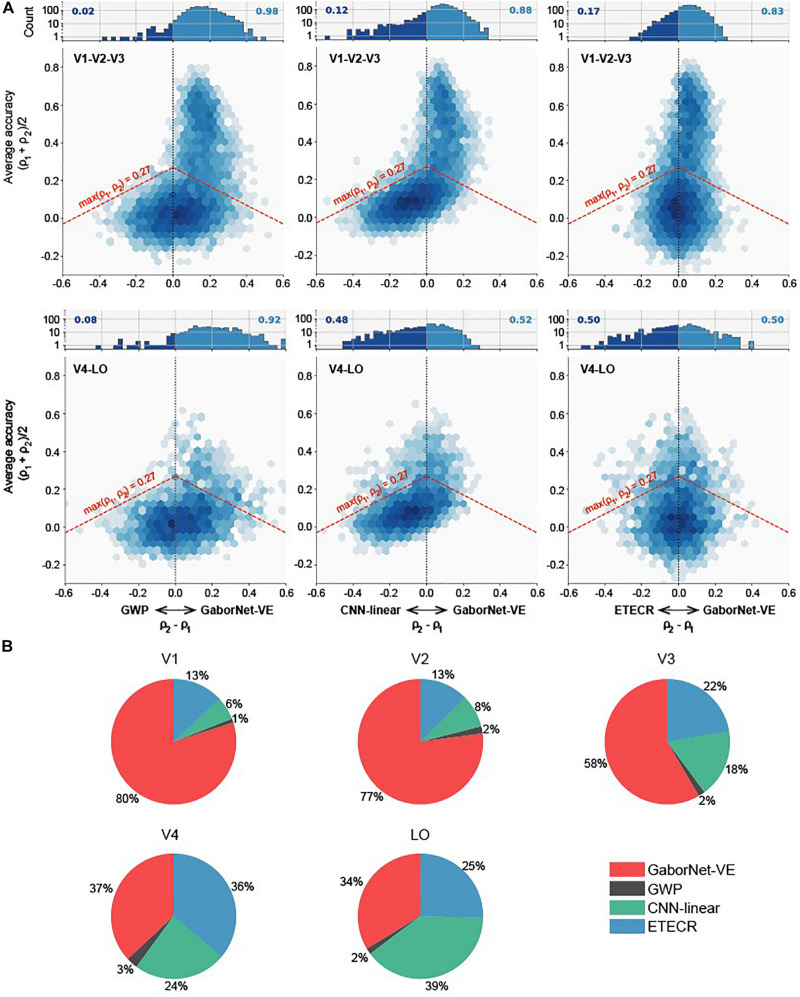
Comparisons of GaborNet-VE, GWP, CNN-linear, and ETECR models by sorting voxels in descending order of prediction accuracy. Only accurately predicted voxels are plotted. Red, gray, green, and blue curves represent GaborNet-VE, GWP, CNN-linear, and ETECR models, respectively.

To compare the visual encoding models for a group of voxels, we considered two approaches as follows. In the first approach, a voxel-to-voxel comparison is made for the same voxel among the two compared models. As shown in Figure 2, scatter plots were used for visualizing and clarifying the results. In these plots, each dot represents one individual voxel. Therefore, the best encoding model for a single voxel can be readily identified. Then, the voxels in each interval were counted to construct an estimate of the voxel density in bar plots (see Figure 2). The second approach compares the overall model performance outcomes after sorting all voxels. As shown in Figure 3, line plots were constructed to represent voxels in descending order of the prediction accuracy.

## Results

We trained, evaluated, and then tested the GaborNet-VE model using a subset of a classical dataset, which contained the functional BOLD signals generated in response to viewing grayscale natural images. The signals were generated from 1,294, 2,083, 1,790, 1,535, and 928 voxels in the V1, V2, V3, V4, and LO areas in the ventral visual stream of S1, respectively. The GaborNet-VE model followed a region-based encoding scheme, where all voxels of one ROI are jointly encoded. Therefore, five GaborNet-VE models with different parameters were fit by the stimuli and fMRI data of the five areas.

### Gabor Kernel Visualization and Receptive Field Estimation

**FIGURE 4:**
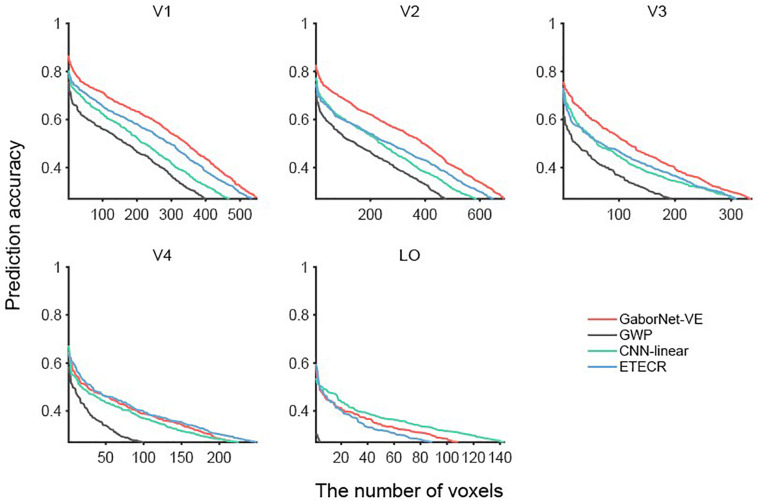
Visualization of the Gabor kernels and RFs. **(A)** Visualization of Gabor kernels. Each of images in first row includes 64 real Gabor kernels learned by real Gabor convolutional layer in GaborNet-VE model for each ROI. Similarly, each of images in second row includes 64 imaginary Gabor kernels learned by imaginary Gabor convolutional layer in GaborNet-VE model for each ROI. **(B)** Visualization of RFs and preferred Gabor kernels. RFs of preferred Gabor kernels for each of five representative voxels are plotted. For each ROI, upper image shows RFs, whereas lower image shows eight preferred Gabor kernels. **(C)** Fitting of RFs to ROIs. Vertical axis represents ratio of RF size to whole image size. Horizontal axis represents five ROIs. **(D)** Tuning of Gabor kernel parameters to ROIs. Vertical axis represents parameter means for preferred Gabor kernels and top 100 best voxel predictions. Blue, orange, green, and red curves represent means of parameters ω, σ, φ, and θ, respectively. Horizontal axis represents five ROIs.

The Gabor kernels in the first convolutional layer of each of the five constructed GaborNet-VE models are visualized in Figure 4A. It is quite challenging to analyze the similarities and differences of the five visual areas from the Gabor kernel maps and to find out which Gabor kernel plays an essential role for specific voxels. Therefore, we implemented the GBP algorithm to back-propagate from the top 100 voxels with the best predictions in each visual area to obtain the RF on the stimulus image and eight preferred Gabor kernels for each voxel. For convenience and space limitations, only five representative voxels were selected from the 100 voxels of each visual area to visually summarize the overall results (see Figure 4B). In Figure 4B, “Vol.” is defined as a form of the serial number of a voxel. For V1, the RFs of most voxels are concentrated in a small region, and these voxels preferred Gabor kernels with smaller scales and higher spatial frequencies (e.g., Vol. 5, Vol. 178, Vol. 689, and Vol. 826). However, a small number of voxels preferred larger Gabor kernels with lower spatial frequencies (such as Vol. 870, which has a significantly large RF). For V2, some voxels exhibited RFs similar to those of V1 (e.g., Vol. 363 and Vol. 383), whereas most V2 voxels show larger concentrated RFs than those of the V1 voxels. The preferred Gabor kernels of the V2 voxels (such as Vol. 300 and Vol. 321) have larger scales and lower spatial frequencies than those of the V1 voxels. However, a few voxels prefer Gabor kernels with larger scales and lower spatial frequencies (such as Vol. 490, whose RF is relatively large and more scattered). For V3, most voxels have larger RFs than those of the V2 voxels, but some of these V3 voxels (such as Vol. 65 and Vol. 81) are concentrated, and some others (such as Vol. 1445) are dispersed. There are a few V3 voxels, such as Vol. 284 and Vol. 410, with similar RFs to those of the voxels in V1 or V2. In addition to the irregular pattern of the RFs, the preferred Gabor kernels of the voxels in V3 are also more complex, with various scales and spatial frequencies. For V4, many voxels (such as Vol. 111 and Vol. 132) have larger concentrated RFs than those of the voxels in V3. Also, there are a few V4 voxels, such as Vol. 1397, with small RFs such as those of the voxels in V1 or V2. There are voxels, such as Vol. 257, with RFs that cover most of the image and that begin to appear for the first time in V4. For LO, there are some voxels (e.g., Vol. 76 and Vol. 145) with large RFs such as those of the voxels in V4. Other LO voxels (e.g., Vol. 374 and Vol. 427) have small RFs such as those of the voxels in V1 or V2, whereas a few voxels (e.g., Vol. 253) have RFs that cover almost the whole image. The preferred Gabor kernels of the voxels in V4 and LO are similar, and these kernels mostly have large scales and low spatial frequencies.

In summary, most of the voxels in V1 and V2 have small concentrated RFs, whereas most of the voxels in V4 and LO have large concentrated RFs. The preferred Gabor kernels of most of the voxels in V1 and V2 have small scales and high spatial frequencies, whereas the kernels of most of the voxels in V4 and LO have large scales and low spatial frequencies. However, a few voxels oppose these conclusions for V1, V2, V4, and LO. Moreover, the voxels in V3 are capricious, regardless of the size of the RF and the properties of the preferred Gabor kernels. To show the overall results more clearly, we have computed statistics for the results of the top 100 voxels of each area. The average ratio of the RF size to the entire image size is plotted in Figure 4C. Obviously, the RF size becomes larger as we go from V1 to V4 but decreases as we go from V4 to LO. The means of the four parameters (ω,σ,φ,θ) of the preferred Gabor kernels are displayed in Figure 4D. Clearly, the mean of ω has an overall downward trend (Mann–Kendall test, *p* < 0.05) as we go from V1 to LO (the blue line in Figure 4D), whereas the mean of σ has an obvious upward trend (Mann–Kendall test, *p* < 0.05) (the green line in Figure 4D).

### Voxel-to-Voxel Comparisons Between the GaborNet Visual Encoding Model and the Reference Models

Firstly, we compared the encoding performance of the GaborNet-VE model with that of the GWP model, the CNN-linear model, and the ETECR model, respectively. As shown in Figure 2A, this is a voxel-to-voxel comparison based on the prediction accuracy of the corresponding voxels. Then, the dominant voxels of each of the two compared models were counted (Figure 2A). Finally, we carried out a voxel-to-voxel comparison of the four models and calculated the ratio of the voxels best predicted by each model. In general, the prediction accuracies of the four models for the low-level visual areas (the first row of Figure 2A) are higher than those of the intermediate- and high-level visual areas (the second row of Figure 2A). The highest prediction accuracy in the low-level visual areas exceeds 0.8, whereas the highest prediction accuracy in the intermediate- and high-level visual areas is only approximately 0.6. Combining Figures 2A,B, we demonstrate that the GaborNet-VE model has the strongest overall performance for the low-level visual areas. The percentages of the dominant voxels of the GaborNet-VE model in comparison with each of the three other models are 98, 88, and 83%, respectively (Figure 2A). Also, the percentages of the dominant voxels of the GaborNet-VE model in comparison with all of the three other models are 80, 77, and 58% in V1, V2, and V3, respectively. For the intermediate- and high-level visual areas, the GaborNet-VE prediction performance has a strong advantage over the GWP model and is almost equivalent to the performance of each of the other two models. Specifically, the percentages of the dominant voxels of the GaborNet-VE model in comparison with each of the three other models are 92, 52, and 50%, respectively (Figure 2A). As well, the percentages of the dominant voxels of the GaborNet-VE model in comparison with all of the three other models are 37 and 34% in V4, and LO, respectively. Hence, the performance of the GaborNet-VE model has an overall downward trend as we go from V1 to LO. From the perspective of voxel distribution, “banana” shapes are shown in the four diagrams in the first two columns of Figure 2A, especially in the upper-middle diagram. These shapes indicate that voxels with high accuracy are mostly on the side of the GaborNet-VE model, whereas voxels with low accuracy are mostly on the side of the reference models. Therefore, voxels with low prediction accuracy under the reference models are effectively rescued by our GaborNet-VE model. Also, these “banana” shapes demonstrate that the GaborNet-VE model provides effective regularization, which benefits from the selective voxel optimization strategy. Because the ETECR model used the same optimization strategy, the voxel distribution of the two diagrams in the last column shows a highly linear pattern (Figure 2A).

### Model Comparison Based on Voxel Sorting by Prediction Accuracy

Voxels whose responses could be accurately predicted by each of the four models (ρ > 0.27) were selected and then sorted in descending order of the prediction accuracy (see Figure 3). Overall, results of the GWP model (the gray lines in Figure 3) are significantly worse than those of the GaborNet-VE model (the red lines in Figure 3), the ETECR model (the blue lines in Figure 3), and the CNN-linear model (the green lines in Figure 3). Therefore, we focus on comparing the GaborNet-VE model only with the CNN-linear model and the ETECR model. The numbers of voxels in V1, V2, V3, V4, and LO, which are accurately predicted by the GaborNet-VE model, are 548, 688, 336, 223, and 108, respectively. These numbers correspond to voxel percentages of 42.35, 33.03, 18.77, 14.53, and 11.64%, respectively. In the first row of Figure 3, the red line is absolutely above the green and blue lines. This demonstrates that the GaborNet-VE model is definitely better than the ETECR and CNN-linear models in predicting the responses in the primary cortex (V1, V2, and V3). However, in the left figure in the second row, the red, green, and blue lines are intertwined. This indicates that the prediction performance outcomes of the three models for the intermediate visual area (V4) are almost equivalent. In the right figure in the second line, the green line is slightly above the red and blue lines. This indicates a marginal advantage of the CNN-linear model for the LO area. However, regardless of the model selection, the numbers of voxels accurately predicted in V4 and LO are much smaller than those in V1, V2, and V3.

## Discussion

We have introduced the GaborNet-VE model, a new end-to-end region-based encoding model for the brain visual ventral stream. The key design principle of this model is constructing a more effective non-linear feature space to fit the brain BOLD responses through learning deep features adapted from handcrafted features. This design was realized by replacing regular convolutional kernels with Gabor kernels with learnable parameters in the first convolutional layer. This combination of the handcrafted and deep features was used to improve the GaborNet-VE model in terms of both expressiveness and biological interpretability, which are viewed as the two most important performance goals of visual encoding models. As the results of this work demonstrated, the lightweight encoding model, composed only of three convolutional layers and one fully connected layer, achieved better prediction accuracy than other compared models for most of the voxels in the visual ventral stream. Moreover, the visualization results showed that the GaborNet-VE model could precisely depict the RF of each voxel and reveal the ROI regularity of fitting to visual features.

### Relationship to Previous Work

Although the GaborNet-VE model is an end-to-end model, it can be mapped into the two steps of linearizing encoding models (Naselaris et al., 2011). The three convolutional layers of GaborNet-VE with the ReLU activation function represent the non-linear feature mapping from the input space to the feature space, whereas the fully connected layer represents the linear mapping from the feature space to the activity space. Therefore, the GaborNet-VE model is a special case of the linearizing encoding models, which fits the non-linear feature mapping and the linear mapping together. Several important models have preceded the GaborNet-VE model. One classical model is the GWP model (Kay et al., 2008), which broke through the bottleneck of visual encoding. However, this encoding model is based on handcrafted features, has limitations on feature variations, and hence has poor expressiveness and universality. The GaborNet-VE model could be viewed as an improved variant of the GWP model, where the fixed-parameter Gabor filters were replaced by Gabor filters with learnable parameters, and the non-linear energy mapping was replaced by two convolutional layers with ReLU activation functions. Another widely known important class of models is that of the CNN-linear models (Güçlü and van Gerven, 2015; Eickenberg et al., 2016), which can obtain abundant features to represent voxel responses. However, many unsuitable features were introduced due to the data–task mismatch of this task-driven approach. Moreover, the models get easily trapped into local optima because the models still have two independent steps. The ETECR model addressed the same type of visual encoding problems (Qiao et al., 2019), but it lacks sufficient biological interpretability. The GaborNet-VE model evolved from the ETECR model, whose first convolutional layer was replaced by a parameterized Gabor convolutional layer. The structured features obtained by Gabor kernels improved the interpretability of the end-to-end model.

### Advantages of the GaborNet Visual Encoding Model

The GaborNet-VE model presented here follows an effective visual encoding methodology in which an adequate non-linear feature space can be constructed by an end-to-end training process based on the statistics of the visual input and the brain BOLD activities. This neurophysiologic and computational approach accumulates several earlier enhancements and has many advantages in encoding the brain ventral visual pathway. Firstly, the GaborNet-VE model has good expressiveness. The results of this work showed that the encoding model had achieved the state-of-the-art prediction performance for the primary visual cortex, and it is also of comparable prediction performance for the intermediate- and high-level cortex areas. We concluded that the high prediction accuracy could be ascribed to the following reasons: (a) the non-linear feature space learned based on the Gabor features represents the voxel responses in low-level areas; (b) the end-to-end training scheme boosted the performance of the proposed model through learning all optimal parameters simultaneously; (c) the selective optimization strategies for features and voxels led to highly effective features and voxels. Secondly, the GaborNet-VE model has good biological interpretability. The encoding model could learn the relatively precise voxel RFs, rather than roughly measuring the RF size by the feature-weighted RF approach (St-Yves and Naselaris, 2018). As the results suggest, voxels in early visual areas have smaller RFs, whereas voxels further along the ventral pathway have larger RFs. This is consistent with some early findings (Hubel and Wiesel, 1962; Gross et al., 1971; Naselaris et al., 2011). Moreover, the model could learn for each voxel the preferred Gabor kernels, whose properties demonstrated the ROI regularity of tuning to visual features. Overall, the voxels in V1 and V2 prefer the Gabor kernels with high spatial frequencies, whereas the voxels in V4 and LO prefer the Gabor kernels with low spatial frequencies, although few voxels may have opposing preferences in V1, V2, V4, and LO. This phenomenon is consistent with the results of one electrophysiology study, which surprisingly found neurons with low spatial frequencies in V1 and neurons with high spatial frequencies in V4 (Lu et al., 2018). Thirdly, the GaborNet-VE model is highly efficient. The previous voxel-wise encoding models fit one linear regression model for each voxel. Eventually, thousands of encoding models need to be fit for several ROIs. However, the GaborNet-VE model uses an ROI-wise scheme, where all voxels of one ROI are jointly encoded. Therefore, the GaborNet-VE model acts as a single model that needs to be trained for an ROI. Fourthly, the GaborNet-VE model is a lightweight model. This model merely consists of three convolutional layers and one fully connected layer, whose total number of layers is four. Generally, CNN-based visual encoding modeling approaches can be categorized into either task- or data-driven approaches. In terms of task-driven approaches, AlexNet with eight layers is the most lightweighted DNN. Also, the parameter number of AlexNet is approximately 60M; however, that of GaborNet-VE is approximately 10K. In terms of data-driven approaches, the ETECR model is the most lightweighted DNN, which has five layers. Moreover, we replaced regular convolutional kernels with Gabor convolutional kernels in the first layer, further reduced the number of parameters to be learned. Fifthly, the GaborNet-VE model is relatively robust. It is generally accepted that the network robustness will improve after the regular convolutional kernels are replaced by the structured Gabor kernels.

### Limitations and Future Directions

Although the GaborNet-VE model has multiple key advantages, this model still has several disadvantages. The results suggest that the prediction performance of that model for the intermediate and high-level areas is still unsatisfactory (see Figures 2, 3). We noticed as well that the GaborNet-VE model is easily prone to overfitting during training, although the model is lightweight. On the other hand, voxels in high-level visual areas depend on a more complex feature representation, which requires a network with more layers. Therefore, a large fMRI dataset is crucially needed. A common problem of current visual encoding models is that they cannot accurately express neural activities in higher visual areas due to the limited fMRI samples. To weaken the influence of data scarcity, we can implement advanced few-shot deep learning and meta-learning techniques, such as the meta-learning approach. The GaborNet-VE model has both good expressiveness and biological interpretability, and this validates the advantages of combining deep and handcrafted features. Therefore, a future research direction should address how to combine deep network features with handcrafted ones more effectively.

## Conclusion

We have proposed the GaborNet-VE model, a new end-to-end ROI-wise encoding model for the brain ventral visual stream. The results of this study demonstrated that the GaborNet-VE model achieved state-of-the-art prediction performance for the primary visual cortex. Hence, the deep-network feature space learning based on Gabor features is more expressive and effective. Moreover, the GaborNet-VE model has good biological interpretability, which could be demonstrated by the ROI regularity of tuning to visual features and the visualization of the estimated RFs. Hence, the proposed lightweight visual encoding model has both good expressiveness and biological interpretability based on combining deep and handcrafted features.

## Data Availability Statement

The datasets presented in this study can be found in online repositories. The names of the repository/repositories and accession number(s) can be found below: http://crcns.org/data-sets/vc/vim-1.

## Ethics Statement

The studies involving human participants were reviewed and approved by the UC Berkeley Office for Protection of Human Subjects (cphs.berkeley.edu). The patients/participants provided their written informed consent to participate in this study.

## Author Contributions

YC, KQ, and LW performed the conceptualization and investigation. YC and KQ performed the data curation, formal analysis, and methodology. YC, KQ, and CZ performed the software. CZ, LW, BY, and LT performed the validation. YC, KQ, CZ, LW, and BY performed the visualization and writing – original draft. LT provided article review and assistance with the final article write up. BY and LT performed the funding acquisition, supervision, and project administration. All authors have read and agreed to the published version of the manuscript.

## Conflict of Interest

The authors declare that the research was conducted in the absence of any commercial or financial relationships that could be construed as a potential conflict of interest.
